# Supplementary material for: Food & You: A digital cohort on personalized nutrition
Source: PLOS Digit Health. 2023 Nov 30;2(11):e0000389. doi: 10.1371/journal.pdig.0000389 (PMC10688868; doi:10.1371/journal.pdig.0000389)
Supplement: S1 Table — (PDF) [file pdig.0000389.s001.pdf]

Inclusion Criteria	B Cohort	C Cohort
Aged over 18	✓	✓
Swiss residency	✓	✓
To own a NFC-enabled smartphone	✓	✓
French German or English speaking	✓	✓
To take part in the project only once	✓	✓
Not in pregnancy	✓	✓
Not in dialysis	✓	✓
Not taking immunosuppressive medication	✓	✓
Not breastfeeding	n.a.	✓
No antibiotic intake in the last thee months	✓	✓
Absence of chronic gastrointestinal disorder	✓	✓
Absence of active inflammatory or neoplastic disease in the last 3 years	✓	✓
Absence of neuropsychiatric disorder	✓	✓
Absence of myocardial infarction or cerebrovascular accident in the last 6 months	✓	✓
Absence of (pre-) diagnosis for type 1 or 2 Diabetes Mellitus	✓	✓
No hormonal contraceptive intake	n.a.	✓
No amenorrhea	n.a.	✓
No (pre-) menopaused	n.a.	✓
No abortion in the last 3 months	n.a.	✓
