# Supplementary material for: Food & You: A digital cohort on personalized nutrition
Source: PLOS Digit Health. 2023 Nov 30;2(11):e0000389. doi: 10.1371/journal.pdig.0000389 (PMC10688868; doi:10.1371/journal.pdig.0000389)
Supplement: S2 Table — (PDF) [file pdig.0000389.s002.pdf]

**Diet**	**Days 2-3 (*16-17)**	**Days 4-5 (*18-19)**	**Days 6-7 (*20-21)**
Normal	110g white bread	110g white bread with 30g butter	50g glucose
Non-dairy	110g white bread	55g white bread with 55g dark chocolate	50g glucose
Non-gluten	110g gluten-free white bread	110g gluten-free white bread with 30g butter	50g glucose
Non-dairy-gluten	110g gluten-free white bread	55g gluten-free white bread with 55g dark chocolate	50g glucose

* Additional tracking period for cohort C participants
