# Supplementary material for: Food & You: A digital cohort on personalized nutrition
Source: PLOS Digit Health. 2023 Nov 30;2(11):e0000389. doi: 10.1371/journal.pdig.0000389 (PMC10688868; doi:10.1371/journal.pdig.0000389)
Supplement: S3 Table — Data weighted according to each unique combination of season, day of the week, and individual. Weights were computed as reciprocals of the frequency of each unique combination in the dataset, ensuring a balanced representation of less frequent groupings. Column “Fruits and Vegetables” shows fraction of participants eating at least 5 portions of fruits and vegetables per day, in percent. (PDF) [file pdig.0000389.s003.pdf]

Category	Energy (kcal)	Meat (g)	Dairy (g)	Water (g)	Fruits and Vegetables > 5 (%)	n Observations
total	2205.19 (728.13)	91.99 (111.83)	124.81 (145.81)	965.06 (954.96)	6.81	1013
female	2066.8 (638.03)	81.63 (101.98)	116.77 (134.86)	967.76 (1006.26)	7.04	568
male	2390.87 (797.05)	105.9 (122.46)	135.59 (158.71)	961.45 (881.56)	6.52	445
18-34	2226.85 (757.97)	81.21 (113.8)	121.05 (150.4)	1017.16 (910.85)	4.85	412
35-49	2228.07 (709.53)	101.6 (112.6)	120.34 (138.13)	948.41 (796.66)	5.95	370
50-64	2131.81 (706.14)	97.37 (105.85)	133.74 (148.01)	913.28 (1294.08)	9.66	207
65+	2076.04 (612.48)	82.23 (95.8)	186.8 (148.76)	754.72 (541.56)	29.17	24
german	2224.47 (733.05)	86.18 (109.82)	132.64 (150.85)	1079.26 (1087.83)	8.01	549
latin	2183.18 (721.93)	98.63 (113.73)	115.86 (139.32)	834.69 (755.25)	5.39	464
female.18-34	2074.62 (645.9)	70.64 (99.77)	108.97 (127.6)	1003.57 (856.08)	5.93	253
female.35-49	2109.16 (626.29)	91.25 (104.05)	122.13 (136.06)	936.19 (732.74)	5.76	191
female.50-64	1988.94 (639.18)	91.48 (101.86)	121.65 (145.69)	959.38 (1581.11)	9.32	118
female.65+	1801.92 (450.93)	43.71 (67.55)	182.75 (155.41)	624.63 (435.9)	50.0	6
male.18-34	2481.73 (857.1)	98.91 (132.21)	141.26 (180.56)	1039.92 (995.77)	3.14	159
male.35-49	2362.39 (771.58)	113.28 (120.51)	118.32 (140.44)	962.21 (863.23)	6.15	179
male.50-64	2331.43 (746.18)	105.6 (110.75)	150.64 (149.67)	848.86 (718.57)	10.11	89
male.65+	2163.9 (632.34)	94.58 (100.32)	188.1 (147.05)	796.42 (566.23)	22.22	18
