# Supplementary figures and images for: Food & You: A digital cohort on personalized nutrition
Source: PLOS Digit Health. 2023 Nov 30;2(11):e0000389. doi: 10.1371/journal.pdig.0000389 (PMC10688868; doi:10.1371/journal.pdig.0000389)

a)

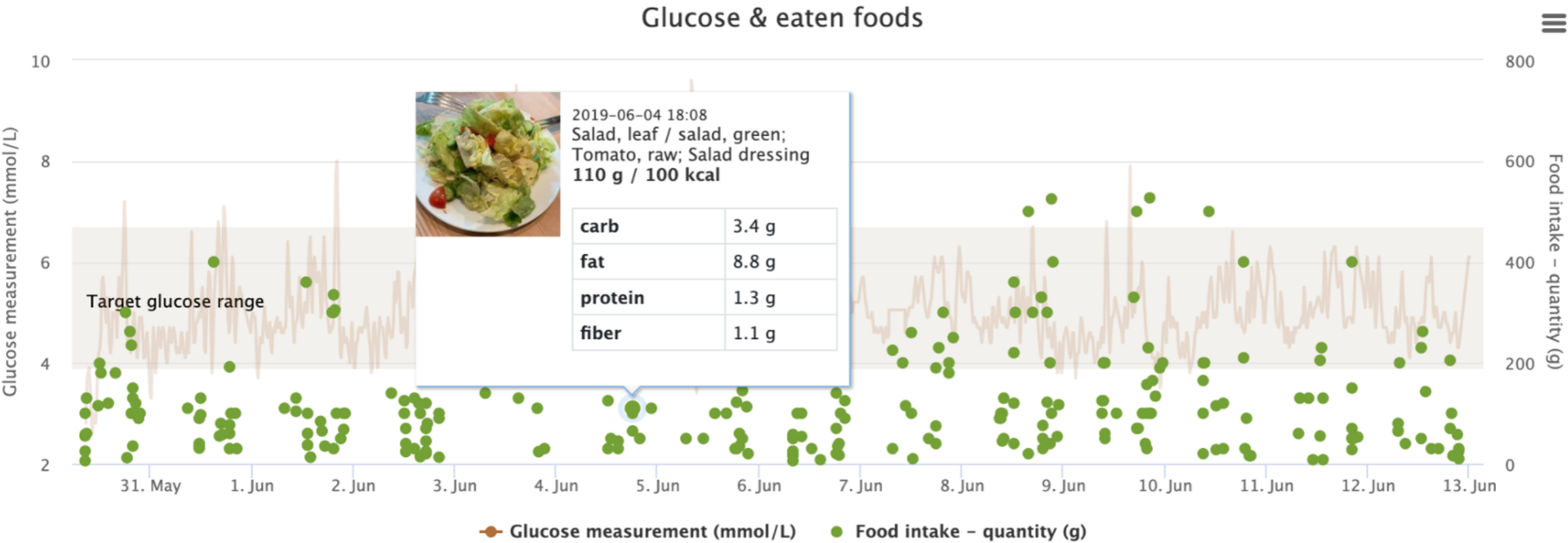

b)

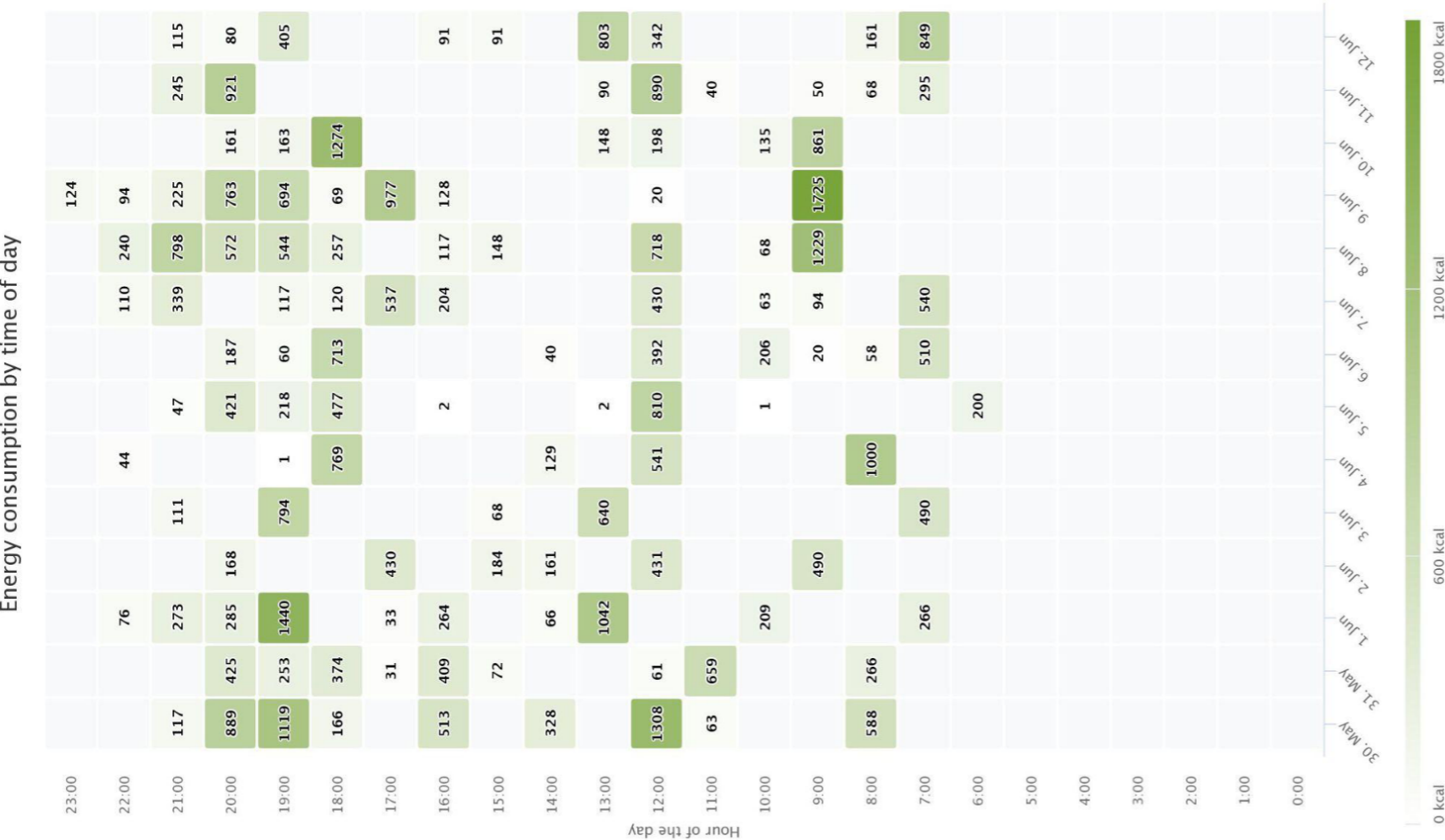

Supplement: S1 Fig — a) Glucose response overlayed with annotated dishes. b) Energy consumption by day. (PDF) [file pdig.0000389.s004.pdf]

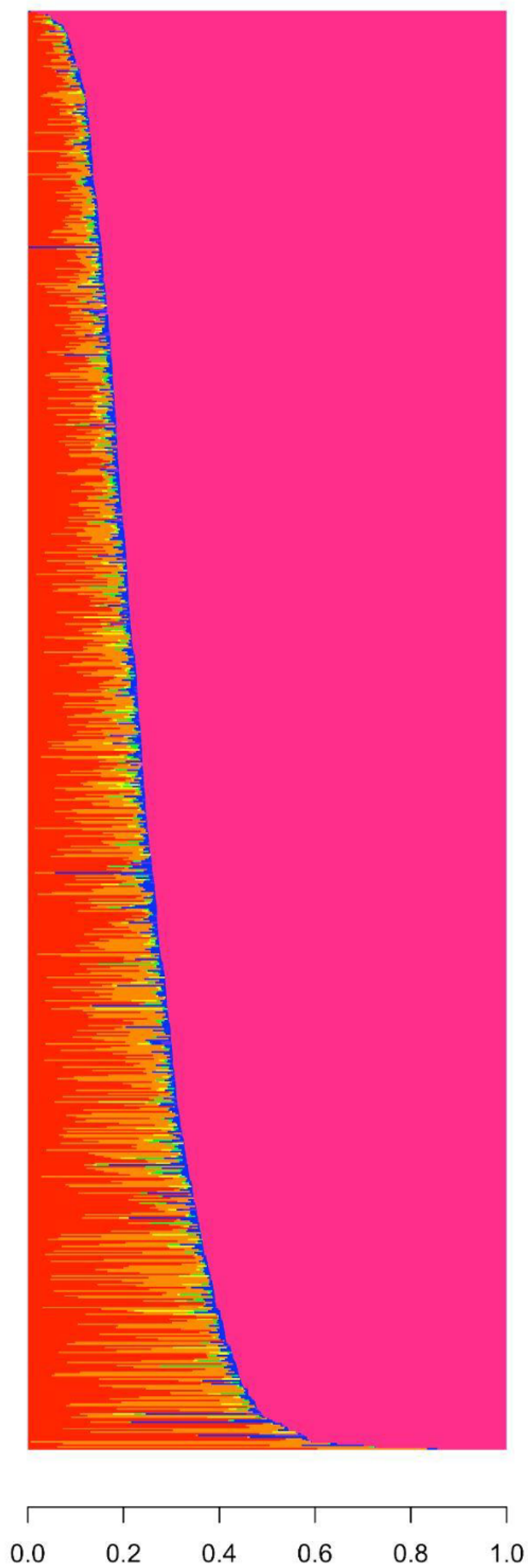

Supplement: S2 Fig — (PDF) [file pdig.0000389.s005.pdf]
