# Supplementary material for: Food & You: A digital cohort on personalized nutrition
Source: PLOS Digit Health. 2023 Nov 30;2(11):e0000389. doi: 10.1371/journal.pdig.0000389 (PMC10688868; doi:10.1371/journal.pdig.0000389)
Supplement: S3 Fig — (PDF) [file pdig.0000389.s006.pdf]

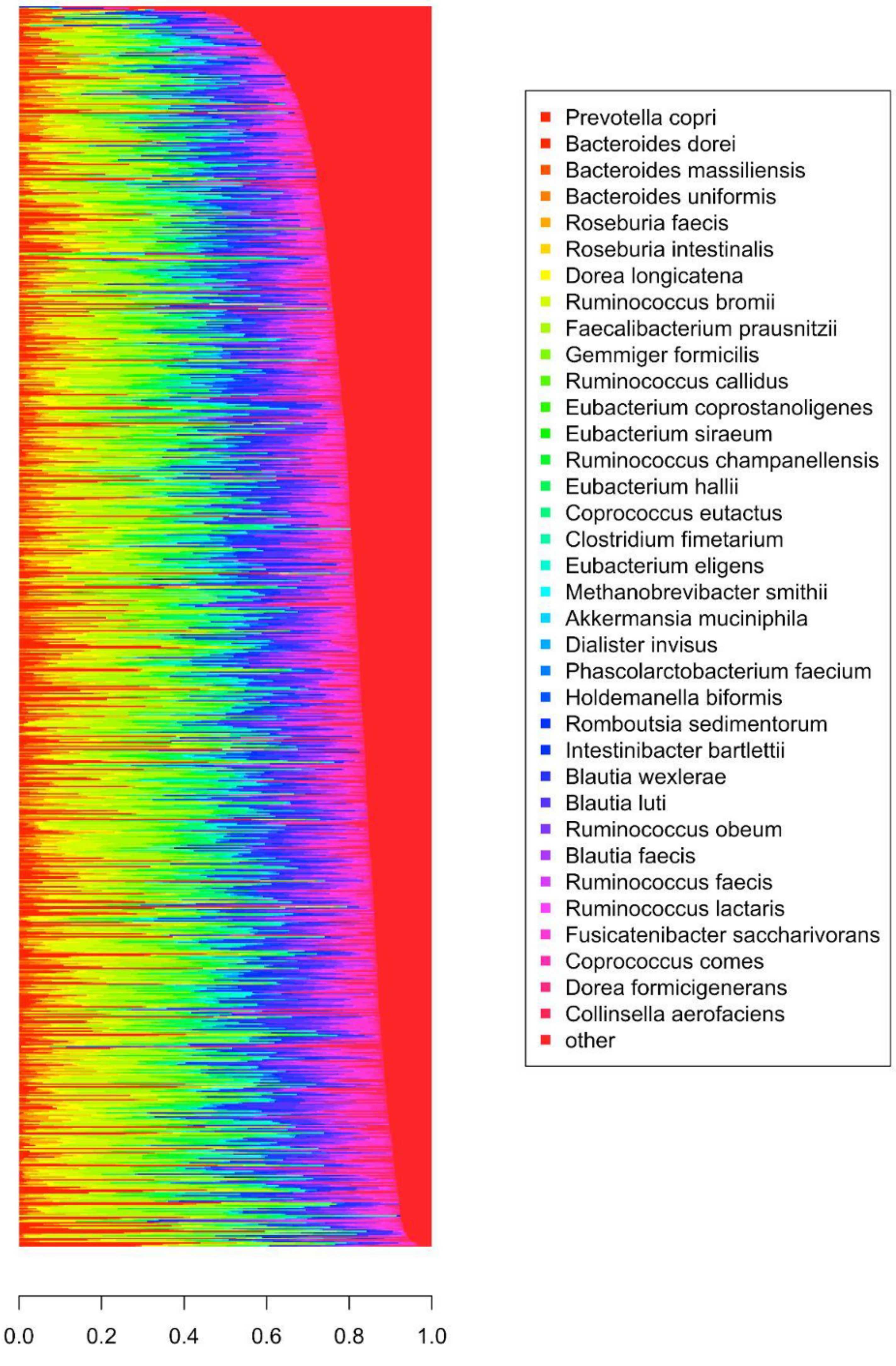
Prevotella copri
Bacteroides dorei
Bacteroides massiliensis
Bacteroides uniformis
Roseburia faecis
Roseburia intestinalis
Dorea longicatena
Ruminococcus bromii
Faecalibacterium prausnitzii
Gemmiger formicilis
Ruminococcus callidus
Eubacterium coprostanoligenes
Eubacterium siraeum
Ruminococcus champanellensis
Eubacterium hallii
Coprococcus eutactus
Clostridium fimetarium
Eubacterium eligens
Methanobrevibacter smithii
Akkermansia muciniphila
Dialister invisus
Phascolarctobacterium faecium
Holdemanella biformis
Romboutsia sedimentorum
Intestinibacter bartlettii
Blautia wexlerae
Blautia luti
Ruminococcus obeum
Blautia faecis
Ruminococcus faecis
Ruminococcus lactaris
Fusicatenibacter saccharivorans
Coprococcus comes
Dorea formicigenerans
Collinsella aerofaciens
other
0.0
0.2
0.4
0.6
0.8
1.0
